# Integrative epigenomic and transcriptomic profiling reveals organ-specific and coordinated cold stress responses in the brain and gill of Nile tilapia

**DOI:** 10.1007/s44154-025-00277-y

**Published:** 2026-01-11

**Authors:** Xinwen Li, Siyao Zhan, Xu Fan, Wei Li, Minghao Zhang, Yu Liu, Mingli Liu, Qihui Wu, Jiulin Chan, Zhichao Wu, Songqian Huang, Liangbiao Chen, Peng Hu

**Affiliations:** 1 Key Laboratory of Exploration and Utilization of Aquatic Genetic Resources, Ministry of Education, Shanghai Ocean University, Shanghai, 201306 China; 2 International Research Center for Marine Biosciences, Ministry of Science and Technology, Shanghai Ocean University, Shanghai, 201306 China; 3 Center for Aquacultural Breeding Research, Shanghai Ocean University, Shanghai, 201306 China; 4 Marine Biomedical Science and Technology Innovation Platform of Lin-Gang Special Area, Shanghai, 201306 China; 5 Shanghai Key Laboratory of Anesthesiology and Brain Functional Modulation, Clinical Research Center for Anesthesiology and Perioperative Medicine, School of Medicine, Translational Research Institute of Brain and Brain-Like Intelligence, Shanghai Fourth People’s Hospital, Tongji University, Shanghai, 200434 China

**Keywords:** Cold stress, Chromatin accessibility, Transcriptional regulation, Epigenetic transcriptional adaptation, Nile tilapia

## Abstract

Cold stress is a major environmental challenge limiting the survival and productivity of tropical aquaculture species such as Nile tilapia (*Oreochromis niloticus*). The brain and gill represent two key organs that orchestrate systemic and environmental responses: the brain serves as the central thermosensory integrator and neuroendocrine control center, while the gill serves as the primary interface for respiration, ion regulation, and immune defense. However, the molecular mechanisms underlying their tissue-specific and potentially coordinated responses to cold remain unclear. Here, we applied integrative ATAC-seq and RNA-seq analyses to systematically investigate chromatin accessibility and gene expression dynamics in tilapia brain and gill tissues under cold stress. We identified thousands of differentially expressed genes and accessible regions, with significant correlations between transcriptional changes. Transcription factor footprinting revealed that Fra1 and Nrf act as key tissue-specific regulators, governing immune, apoptotic, and metabolic reprogramming in the brain and gill, respectively. Notably, the Fra1 module in the brain activated signaling pathways associated with stress response, neurodevelopment, and metabolic regulation which may influence peripheral responses by coordinating systemic physiological adjustments under cold stress, while Nrf-mediated regulation in the gill supported local homeostasis through redox and transport-related mechanisms. These findings highlight the hierarchical and organ-specific transcriptional control underlying cold adaptation in ectotherms. Our study provides the first chromatin accessibility atlas of cold-responsive regulatory networks across central and peripheral organs in fish, offering mechanistic insight and molecular targets for breeding cold-tolerant aquaculture strains.

**Supplementary Information:**

The online version contains supplementary material available at 10.1007/s44154-025-00277-y.

## Introduction

Cold stress, resulting from acute or prolonged exposure to low temperatures, is a major contributor to mortality in aquaculture species, especially those native to tropical and subtropical regions (Sokolova 2021). As aquatic ectotherms, fish rely on external temperatures to regulate their physiological functions, making temperature a key determinant of metabolic rate and overall biological activity (Liu et al. 2022; Reid et al. 2022). Cold exposure can disrupt essential systems including immune function, apoptosis, ion transport, and energy metabolism (Reid et al. 2022). These effects often occur through organ-specific pathways. In the brain, particularly the hypothalamic preoptic area, cold stress impairs thermosensory signaling and cerebral blood flow, leading to neuronal apoptosis and metabolic dysfunction that compromise behavioral coordination (Kristiansen et al. 2020). In the gills, cold exposure triggers mitochondrial damage and induces pro-apoptotic signaling, weakening their critical roles in respiration, osmoregulation, and immune defense (Wood and Eom 2021). The combined effect of these tissue-specific impairments destabilizes systemic physiological homeostasis and reduces the ability of fish to cope with environmental stress (Reid et al. 2022). Understanding the molecular regulatory networks that mediate these organ-specific cold responses is essential for the development of cold-tolerant aquaculture strains and effective stress-resilience strategies.

Although transcriptomic and metabolomic studies have revealed how cold stress alters membrane fluidity, protein activity, and lipid metabolism, ultimately causing oxidative damage in tissues such as the liver, gills, and brain (Long et al. 2012; Los and Murata 2004; Mendoza 2014; Rossi et al. 2017; Wu et al. 2015), these approaches primarily capture downstream effects and lack insight into the upstream regulatory mechanisms. Emerging evidence indicates that epigenetic regulation, particularly changes in chromatin accessibility, plays a key role in modulating transcriptional responses under environmental stress (Beisaw et al. 2020; Yang et al. 2020). Our previous research has shown that *cis*-regulatory elements are important in cold adaptation in zebrafish and tilapia (Hu et al. 2016, 2015). Furthermore, integrative analyses using ATAC-seq and RNA-seq have successfully identified core transcriptional regulators such as Stat1 and Irf1 in tilapia during cold stress (Huang et al. 2025; Jiao et al. 2025). These multi-omics strategies offer high-resolution insights into regulatory circuits governing cold adaptation in ectothermic vertebrates. However, their application to brain and gill tissues, which are central to neuroendocrine signaling and environmental response, respectively, remains unexplored. Additionally, how transcriptional programs are coordinated across organs during cold exposure in tropical fish remains poorly understood.

Nile tilapia (*Oreochromis niloticus*), the second most widely farmed freshwater fish species globally, contributes over 6 million tons to annual aquaculture production (Geletu and Zhao 2023). Originating from tropical Africa, tilapia has a relatively narrow thermal tolerance range, with a lower threshold typically above 12 °C, which limits its geographic distribution and overwinter survival (Blasco et al. 2022). The brain, particularly the hypothalamus, functions as the neuroendocrine hub regulating hormonal stress responses and circadian rhythms (Gao et al. 2022; Topal et al. 2021). In response to cold, the brain activates the hypothalamic–pituitary axis to adjust cortisol levels, initiating systemic adaptation to temperature decline. In contrast, gill tissues, which are constantly exposed to the external aquatic environment, play vital roles in gas exchange and ion regulation and are directly responsible for maintaining physiological homeostasis during thermal fluctuations (Wen et al. 2018).

In this study, we established an integrative multi-omics framework combining ATAC-seq and RNA-seq to systematically characterize the transcriptional and epigenetic responses to cold stress in Nile tilapia, focusing on the brain and gill as representative organs of central and peripheral regulation. By profiling gene expression dynamics, chromatin accessibility, and transcription factor (TF) activity, we identified both tissue-specific cold-responsive TFs and their associated regulatory modules. Integrating differentially accessible chromatin regions with differentially expressed genes enabled the reconstruction of spatially resolved transcriptional regulatory networks. These networks revealed distinct regulatory mechanisms operating in the brain and gill, highlighting divergent utilization of conserved TFs in central versus peripheral cold adaptation. Our findings close an important gap in understanding how chromatin-level changes mediate cold stress responses in fish and provide mechanistic insights to guide the development of cold-tolerant strains for sustainable aquaculture.

## Results

### Transcriptional and chromatin landscape under cold stress

**Fig. 1:**
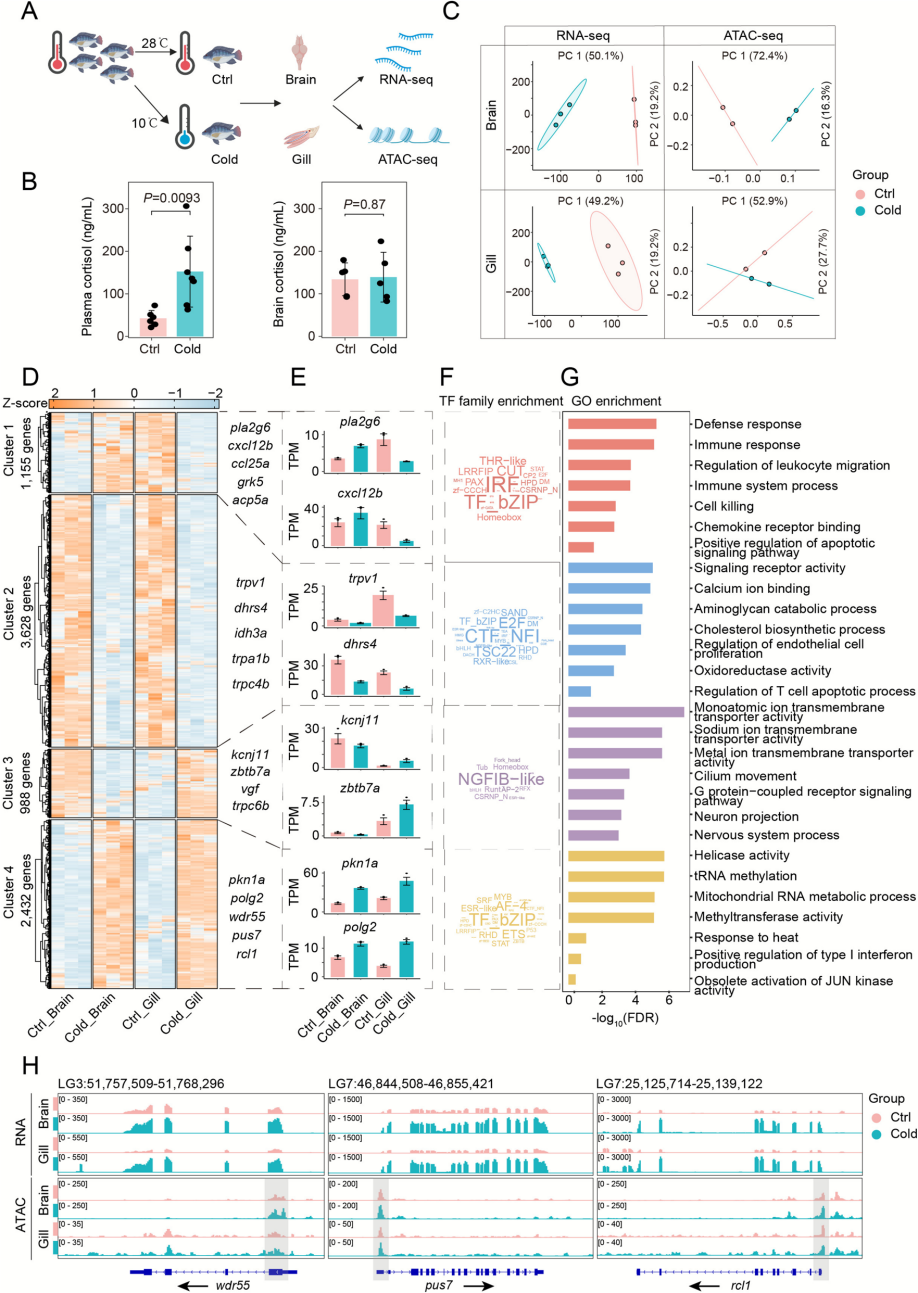
Integrated transcriptomic and epigenomic profiling of tilapia Brain and Gill tissues during cold stress. **A** Experimental workflow for RNA-seq and ATAC-seq analyses to evaluate transcriptional changes and chromatin accessibility under cold stress conditions. **B** Cortisol levels in plasma and brain tissue under control and cold stress conditions. Cortisol concentrations were measured in plasma (n = 7 per group) and brain tissue (n = 5 per group) of Nile tilapia maintained at 28 °C (ctrl) or exposed to 10 °C (cold). Data are presented as mean ± SD. SD, standard deviation. Statistical significance between control and cold-stress conditions within each tissue was determined by *t*-test. **C** Principal component analysis (PCA) of RNA-seq (left) and ATAC-seq (right) data, demonstrating clear separation by tissue type and stress response. **D** K-means clustering of gene expression patterns under cold stress, categorized into four distinct clusters. **E** Representative differentially expressed genes (DEGs) from each cluster, with error bars denoting the standard error (SE) of TPM values across replicates. **F** Enrichment analysis of transcription factor (TF) families associated with clustered DEGs during cold stress. **G** Gene Ontology (GO) term enrichment for clustered DEGs, with statistical significance assessed by FDR. **H** Integrative Genomics Viewer (IGV) browser views of key cold-responsive genes (*wdr55, pus7,* and *rcl1*), with genomic coordinates indicated above each track. PCA, principal component analysis; DEGs, differentially expressed genes; SE, standard error; TPM, transcripts per million; TF, transcription factor; GO, Gene Ontology

To investigate transcriptional and chromatin accessibility dynamics under cold stress and identify core regulators, we generated RNA-seq (12 libraries) and ATAC-seq (8 libraries) data from brain and gill tissues of control and cold-stressed Nile tilapia (*Oreochromis niloticus*) (Fig. 1A). These tissues were selected due to their central roles in coordinating cold stress responses. To physiologically validate the systemic stress response, cortisol levels were measured in plasma and brain tissue following cold exposure. The results demonstrated a significant increase in plasma cortisol levels under cold stress, whereas no significant change was observed in brain tissue (Fig. 1B). This pattern aligns with the established role of cortisol as a primary systemic stress hormone in teleost fish (Mommsen et al. 1999). RNA-seq yielded 27.49–42.07 million reads per sample (Table S1), and ATAC-seq yielded 26.42–62.96 million high-quality reads (Table S2), confirming data robustness. High reproducibility among RNA-seq replicates was observed (Fig. S1A), and principal component analysis (PCA) revealed tissue-specific clustering (PC1) and temperature-induced separation (PC2) (Fig. 1C).

We detected 23,440 expressed genes (TPM > 1; Table S3) and identified 4,643 differentially expressed genes (DEGs) in the brain and 5,610 in the gill under cold stress (fold change > 2, *P*-value < 0.05; Fig. S1B; Table S4). In the brain, 1,951 DEGs were upregulated and 2,692 downregulated; in the gill, 2,213 were upregulated and 3,397 downregulated. Of these, 702 upregulated and 1,033 downregulated genes were shared between tissues (Fig. S1C). Key DEGs—*trpa1b, dhrs11, nfkb2, foxo4,* and *cdkn1a*—were strongly dysregulated and are known to mediate stress, immunity, and apoptosis (Endo et al. 2019; Fathi et al. 2024; Liu et al. 2019; Murphy et al. 2023; York 2023).

KEGG enrichment of shared DEGs indicated pathways such as IL-17 signaling, TRP channel regulation, ferroptosis, arachidonic acid metabolism, and lipolysis in adipocytes. Brain-specific DEGs were enriched in p53, MAPK, GnRH signaling, glutamatergic synapse, and aldosterone synthesis. Gill-specific DEGs were enriched in apoptosis, platelet activation, glutathione metabolism, and DNA-sensing pathways (Fig. S1E).

K-means clustering of DEGs revealed four expression modules (Fig. 1D). Cluster 1 (brain up, gill down) included *pla2g6, cxcl12b, ccl25a, grk5*, and *acp5a*, enriched in immune and apoptosis pathways. TF enrichment pointed to IRF, CUT, and STAT families (Fig. 1F), consistent with immune regulatory functions. GO analysis confirmed enrichment in immune response, leukocyte migration, and cell killing (Fig. 1G). Cluster 2 genes (downregulated in both tissues), such as *jak3, trpv1* and *trpa1b*, were enriched in calcium signaling, cholesterol biosynthesis, and apoptosis regulation, with involvement of CTF_NFI, E2F, and TSC22 TF families. Cluster 3 (brain down, gill up) included *kcnj11* and *trpc6b*, involved in neuronal signaling and ion transport, regulated by NGFIB-like factors. Importantly, TRP channel genes (*trpv1, trpa1b, trpc6b*) emerged as central thermosensors modulating calcium flux (York 2023; York and Zakon 2022). Cluster 4 (upregulated in both tissues), included genes such as *pkn1a, polg2*, and *unc45a*, involved in methylation, interferon response, and DNA repair. We assessed and verified changes in spontaneous calcium influx patterns in HEK293T cells transfected with tilapia *trpv1*, in response to the *trpv1*-specific agonist (Fig. S2).

ATAC-seq showed the expected nucleosomal ladder pattern (Fig. S3) and strong replicate consistency (Fig. S4A). About 66% of accessible chromatin regions localized to promoters, exons, or introns (Fig. S4B). In total, 252,831 peaks were detected, with 20,681 and 2,082 differentially accessible peaks (DAPs) in brain and gill, respectively (Fig. S1B; Table S5). Most DAPs corresponded with DEGs, indicating regulatory relevance. Chromatin accessibility increased in the promoter/genomic regions of cold-induced genes (Fig. 1H), as visualized using IGV for genes such as *wdr55, pus7*, and *rcl1*. These genes showed concurrent upregulation in RNA expression and promoter accessibility, suggesting epigenetically mediated cold-induced transcriptional activation.

**Fig. 2:**
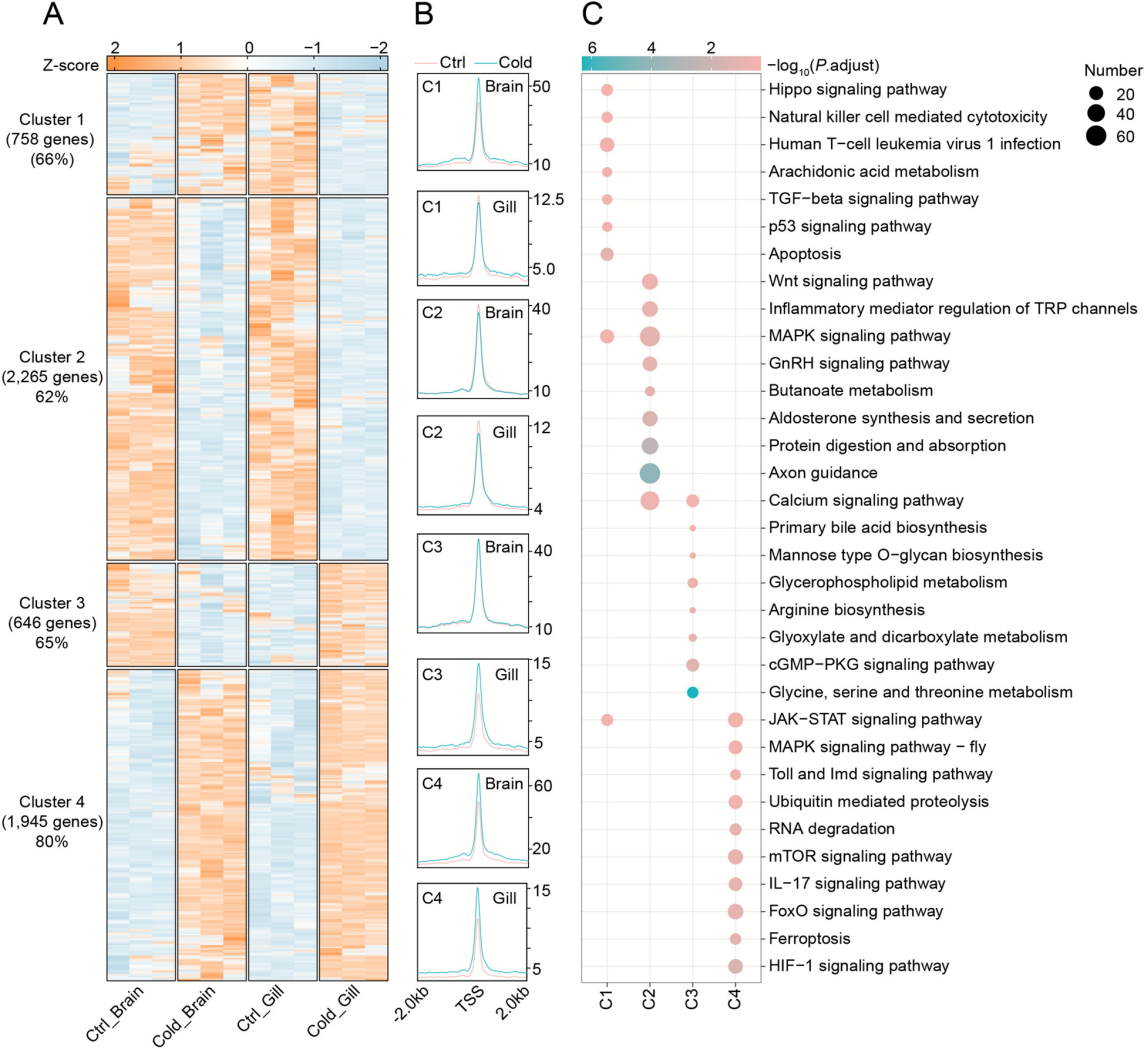
Coordinated chromatin accessibility and transcriptional regulation in tilapia brain and gill tissues during cold stress. **A** Heatmap visualization of expression patterns and chromatin accessibility for 5,614 differentially expressed genes (DEGs), classified into four clusters through k-means clustering. Gene expression levels were z-score normalized, with orange and blue colors denoting up- and down-regulation, respectively. **B** Chromatin accessibility profiles averaged across genomic regions spanning ± 2,000 bp surrounding the transcription start sites (TSS) of the 5,614 DEGs. **C** KEGG pathway enrichment analysis for DEG clusters exhibiting distinct chromatin accessibility changes under cold stress. Color intensity reflects -log10 (*P*. adjust), with statistical significance evaluated by hypergeometric testing (FDR-corrected)

To link chromatin accessibility and transcription, we performed integrated clustering of DEGs and DAPs (Fig. 2A–B; Fig. S5). Approximately 67% of DEGs were co-regulated at the chromatin level. Cluster 1 (brain up, gill down) was enriched for apoptosis, lipid metabolism, and immune signaling. Cluster 2 (down in both tissues) involved metabolism, axon guidance, and calcium signaling. Cluster 3 (brain down, gill up) was associated with lipid and amino acid metabolism. Cluster 4 (up in both tissues) was enriched in RNA degradation, proteolysis, immune signaling, and ferroptosis (Fig. 2C).

### Tissue-specific transcription factor binding dynamics

**Fig. 3:**
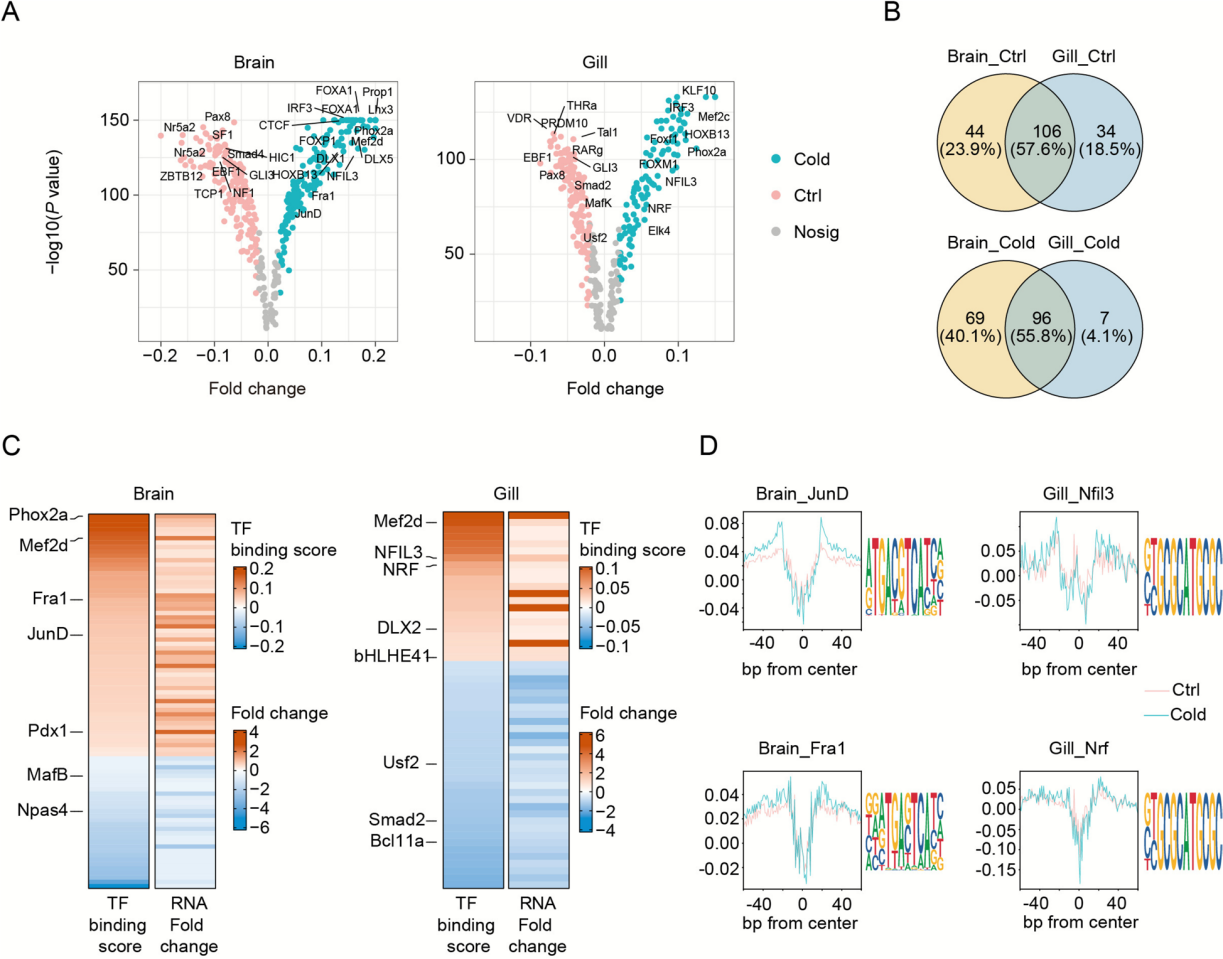
Transcriptional regulatory networks of TFs in tilapia under cold stress. **A** Volcano plots comparing transcription factor (TF) binding activity between Cold and Ctrl groups. The x-axis shows differential binding scores, while the y-axis displays − log10(*P*-value). Left: Brain tissue comparisons; right: Gill tissue comparisons. Red and blue dots represent significantly up-bound TFs in Cold and Ctrl conditions, respectively (gray: nonsignificant). **B** Venn diagram illustrating shared and tissue-specific TFs binding changes in Brain and Gill tissues during cold exposure. **C** Expression patterns of differentially bound TFs in Brain (left) and Gill (right), with color intensity reflecting log2 fold-changes (log2FC) in gene expression. **D** Footprint analysis (left) and corresponding motif sequences (right) for cold-responsive TFs (Fra1/JunD in Brain; Nrf/Nfil3 in Gill). Cold, cold; Ctrl, control; TF, transcription factor; FC, fold change

To systematically identify genome-wide transcription factor binding sites (TFBSs) in response to cold stress, we performed ATAC-seq footprinting analysis using TOBIAS (see Methods). Aggregated footprint plots validated the precision of TF binding predictions. Among the 386 TFs analyzed, 96 TFs exhibited significantly increased binding activity under cold stress, including Fra1, Nfil3, Mef2d, Phox2a, Irf3, and Hoxb13 (Fig. 3A–B).

Footprint scores showed a strong positive correlation with RNA expression in matched samples, even when accounting for motif similarity, supporting the notion that chromatin accessibility changes reflect active transcriptional regulation (Fig. 3C). Notably, transcription factor activity displayed clear tissue-specificity: JunD and Fra1 were predominantly active in brain tissue, while Nfil3 and Nrf exhibited heightened activity in gill (Fig. 3D; Table S7). All of these factors showed deeper footprints under cold stress, indicating enhanced TF occupancy and suggesting a central role in mediating transcriptional responses to low temperature.

Functional annotation provided further insight into the biological roles of these factors. For example, JunD, a component of the AP-1 complex, regulates antiviral immune responses in grass carp (Wan and Su 2015). Fra1, another AP-1 family member, modulates both inflammation and cellular homeostasis (Zeng et al. 2022). Nfil3 is known to regulate immune cell development and apoptosis in teleost fish, including grass carp (Yu et al. 2014). Importantly, cold-induced enhancement of Fra1 and Nrf binding was accompanied by upregulation of their target genes, establishing them as key transcriptional activators involved in cold adaptation in tilapia.

### Regulatory network architecture of cold-responsive transcription factors

To elucidate how cold-responsive *cis*-regulatory elements and trans-acting TFs shape transcriptional regulatory networks (TRNs), we examined TF footprints within accessible chromatin regions, typically spanning 10–50 base pairs (bp). Among the top tissue-specific candidates identified, JunD and Fra1 were prominent in the brain, while Nfil3 and Nrf were selectively enriched in gill tissues (Fig. 3D).

**Fig. 4:**
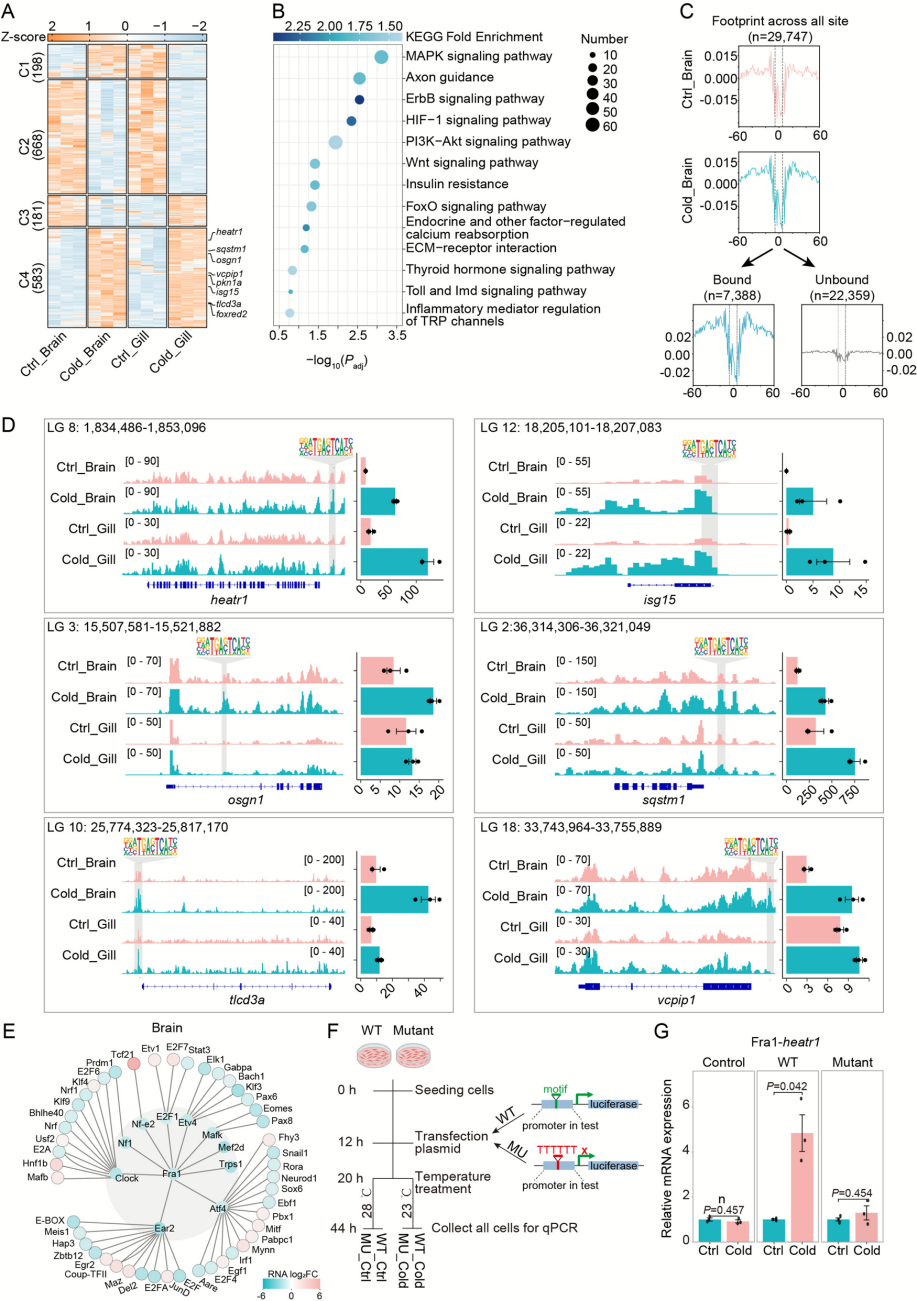
Transcriptional activation by Fra1 through open chromatin binding. **A** Expression patterns of putative Fra1 target genes with promoter or genic binding sites, presented as a z-score normalized heatmap (orange: upregulation; blue: downregulation). **B** KEGG pathway enrichment of Fra1-regulated genes, with statistical significance assessed by hypergeometric testing (FDR-corrected *P*-value), color according to log2FE. **C** Aggregate footprint profiles comparing Fra1 binding (n = 29,747 sites) between Ctrl and Cold conditions (upper panel), with corresponding unbound/bound signals analyzed by TOBIAS (Bentsen et al., 2020) (lower panel). **D** Fra1-mediated transcriptional activation at promoter regions during cold stress. Left: IGV tracks showing Fra1 binding in open chromatin regions of responsive genes (*heatr1, isg15, osgn1, sqstm1, tlcd3a, vcpip1*). Right: Corresponding expression levels (mean TPM ± SE; n = 3 replicates). Genomic coordinates and Fra1 motif locations are indicated. **E** TF regulatory network in Brain tissue, with nodes colored by expression log2 fold-change (Cold vs Ctrl; blue: upregulated, red: downregulated). Cold, cold; Ctrl, control; FDR, false discovery rate; log2FE: log2 Fold Enrichment; SE, standard error; TPM, transcripts per million. **F** Schematic diagram of plasmid constructs for the dual-luciferase reporter assay, depicting the native promoter (top) and the motif-replaced promoter (bottom). **G** The relative mRNA expression levels at 23 °C compared to 28 °C were measured for the wild-type and mutant promoter constructs in EPC cells. Statistically significant differences in mRNA expression between the wild-type and mutant promoters are indicated by *P* values. The empty vector pGL4.23 was used as a control. WT: wild type. MU: Mutant

Fra1, an AP-1 transcription factor known for its role in macrophage activation and regulation of apoptosis and proliferation in mammals (Hannemann et al. 2019), was identified as a key regulator of cluster 4 co-upregulated genes. These targets included *heatr1, isg15, osgn1, sqstm1, tlcd3a*, and *vcpip1* (Fig. 4A). KEGG enrichment analysis revealed that Fra1 target genes were significantly involved in the MAPK signaling pathway, axon guidance, HIF-1 signaling, and FoxO signaling (Fig. 4B). TOBIAS footprinting confirmed that Fra1 binding was strongly enhanced under cold stress (Fig. 4C), and IGV visualization further validated increased chromatin accessibility at Fra1-bound promoters (Fig. 4D), underscoring Fra1’s regulatory influence in the cold-stressed tilapia brain. The TOBIAS-inferred activation network positioned Fra1 at the apex of a hierarchical cascade, directly activating 10 primary TFs (e.g., Clock, Etv4, Ear2, NF-E2, E2F1, Mafk, Atf4, Nf1, Mef2d, Trps1) and 47 secondary TFs in the brian (Fig. 4E; Fig. S6A).

**Fig. 5:**
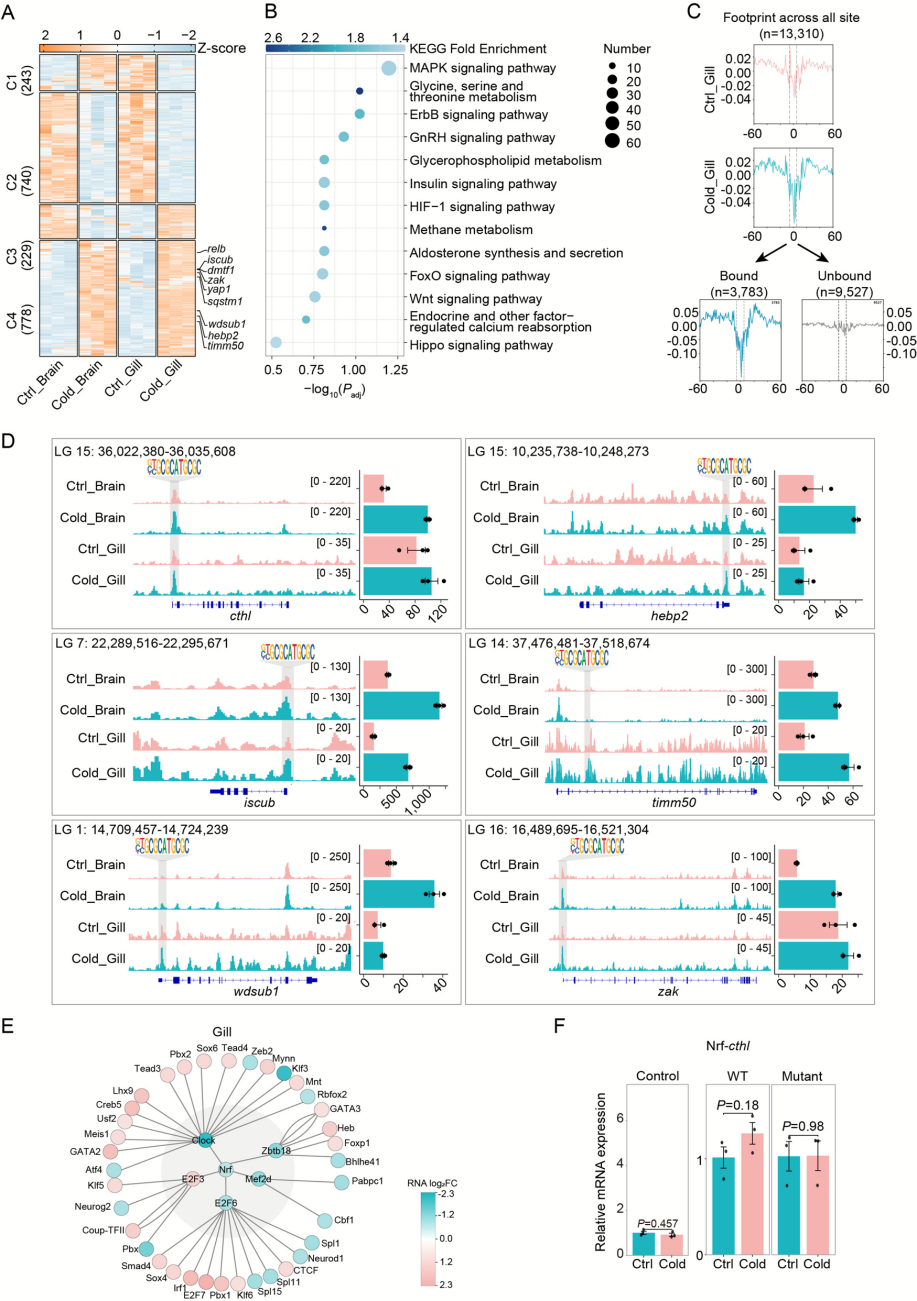
Transcriptional activation by Nrf through open chromatin binding. **A** Heatmap visualization of expression profiles for Nrf target genes exhibiting promoter or genic region binding, with z-score normalized expression levels (orange: upregulated; blue: downregulated). **B** Pathway enrichment analysis (KEGG) of Nrf-regulated genes, with statistical significance evaluated by hypergeometric test (FDR-adjusted *P*-value), color according to log2FE. **C** Aggregate footprint analysis of Nrf binding sites (n = 13,310) under Ctrl and Cold conditions (upper panel), with corresponding bound/unbound signals derived from TOBIAS (Bentsen et al., 2020) analysis (lower panel). **D** Genomic and expression profiles of Nrf targets. Left: IGV tracks displaying Nrf binding in open chromatin regions of responsive genes (*cthl, hebp2, iscub, timm50, wdsub1, zak*). Right: Corresponding transcriptional changes (mean TPM ± SE; n = 3 biological replicates). **E** TF regulatory networks in Gill tissue, with node colors reflecting expression log2 fold-changes (Cold vs Ctrl; blue: upregulated, red: downregulated). FDR, false discovery rate; log2FE: log2 Fold Enrichment; SE, standard error; TPM, transcripts per million; Cold, cold; Ctrl, control. **F** The relative mRNA expression levels at 23 °C compared to 28 °C were measured for the wild-type and mutant promoter constructs in EPC cells. Statistically significant differences in mRNA expression between the wild-type and mutant promoters are indicated by *P* values. The empty vector pGL4.23 was used as a control. WT: wild type

Similarly, Nrf (Nuclear Respiratory Factor), a transcription factor governing oxidative stress, metabolism, and immune homeostasis via antioxidant response elements (AREs) (George et al. 2024; Zhao et al. 2023), was identified as a central regulator in gill tissue. TOBIAS footprinting analysis revealed 1,990 Nrf-bound genes, which were enriched in key pathways such as MAPK signaling, glycine/serine/threonine metabolism, HIF-1 signaling, and glycerophospholipid metabolism (Fig. 5A–B). Under cold stress, Nrf binding was markedly increased (Fig. 5C), with direct target genes including *cthl, hebp2, iscub, timm50, wdsub1*, and *zak*, confirmed through local footprint visualization (Fig. 5D). Pseudotime trajectory analysis further demonstrated that Nrf was positioned upstream of a TRN comprising 5 primary (e.g., Clock, Zbtb18, Mef2d, E2F6, E2F3) and 36 secondary TFs, orchestrating transcriptional reprogramming in the gill during cold exposure (Fig. S6B).

To functionally validate the regulatory potential of the identified Fra1 and Nrf motifs under cold stress, we selected representative target gene pairs for experimental verification: Fra1–*heatr1* and Nrf–*cthl*. Promoter fragments containing either the wild-type motif or a mutant version, in which the core motif sequence was replaced with a poly-thymine tract, were cloned upstream of the firefly luciferase gene in the pGL4.23 vector. These constructs were transiently transfected into EPC (epithelioma papulosum cyprini) cells, which were chosen for their high transfection efficiency, and subsequently incubated at either the control temperature of 28 °C or the cold stress temperature of 23 °C. We quantified the transcriptional changes induced by cold stress by comparing the normalized luciferase activity at 23 °C to that at 28 °C (Fig. 4F). For the Fra1–*heatr1* pair, the wild-type promoter was significantly induced upon cold exposure, whereas this induction was abolished in the mutant construct (Fig. 4G). The Nrf–*cthl* pair also exhibited an upward trend in promoter activity under cold stress, which was attenuated in the mutant, though not to a statistically significant extent (Fig. 5F). These results collectively confirm the functional involvement of the Fra1 motif in the transcriptional response of heatr1 to cold stress and suggest a potential, though less pronounced, role for the Nrf motif in regulating cthl.

## Discussion

Temperature fluctuations are among the most significant environmental stressors affecting fish survival and aquaculture productivity. In tropical aquaculture systems, cold stress represents a major constraint, causing high mortality and limiting the geographic range of economically important species such as Nile tilapia (*Oreochromis niloticus*) (Sokolova 2021). To understand how tilapia adapt to acute thermal fluctuations, particularly in critical organs, this study integrated ATAC-seq and RNA-seq to characterize epigenetic and transcriptional responses in the brain and gills. Our findings reveal coordinated, tissue-specific regulatory programs driven by changes in chromatin accessibility and transcription factor (TF) activity. Notably, Fra1 and Nrf emerged as central regulators in the brain and gill, respectively, mediating cold-responsive transcriptional reprogramming.

In this study, we used a rapid cold-stress condition (28 °C → 10 °C for 12 h) to capture the earliest regulatory events triggered by an acute temperature drop. This temperature represents the critical thermal minimum for our Nile tilapia strain, making it a biologically relevant threshold for examining survival-associated transcriptional and chromatin responses. It is well established that tilapia exhibit highly condition-dependent cold responses: acute severe cooling induces fast chromatin remodeling, whereas prolonged or gradual cooling activates distinct pathways related to cellular damage or long-term acclimation. Therefore, different temperature gradients or durations would likely reveal separate aspects of the cold-response program, while our design specifically targets the acute, early-phase regulatory architecture. Our sampling strategy focused on non-LOE individuals to capture the early molecular programs associated with acute cold tolerance. This design intentionally emphasized fish that were able to maintain equilibrium during the 12-h cold challenge, enabling us to characterize regulatory mechanisms underlying resilience rather than collapse. It is important to recognize that this “tolerant” phenotype is relative and transient; under prolonged or sustained low temperatures, a much larger fraction of the population ultimately exhibits LOE or mortality (El-Sayed et al. 2023). Thus, our dataset reflects the acute-phase responses of a temporarily resistant subgroup rather than fixed tolerant and sensitive categories. A comparative omics analysis between LOE and non-LOE individuals would provide critical insight into the molecular basis of differential cold susceptibility, and we highlight this as an important direction for future investigation.

The brain functions as the central hub for environmental sensing and hormonal regulation, while the gills serve as the primary interface for gas exchange, osmoregulation, and immune defense. Our cortisol measurements further support the proposed model in which the brain coordinates peripheral cold-stress responses through neuroendocrine signaling. Acute cold exposure significantly elevated plasma cortisol levels, while cortisol concentrations in brain tissue remained unchanged, indicating activation of the hypothalamic–pituitary–interrenal (HPI) axis rather than local cortisol production. This pattern is consistent with the well-established role of cortisol as a systemic stress hormone in teleosts (Mommsen et al. 1999). Similar HPI-axis–driven endocrine coordination has been documented in Mozambique tilapia under osmotic stress, where brain activation stimulates cortisol release, which then modulates ion regulatory pathways in the gills (Chang et al., 2023). Extending this framework to cold stress, our findings suggest that the Fra1-associated transcriptional module in the brain may shape downstream gill responses through cortisol-mediated signaling. These results provide physiological evidence for a central-peripheral regulatory axis and highlight cortisol as a key hormonal mediator linking neural sensing with peripheral adaptation during acute cold exposure. These tissues exhibited distinct but complementary transcriptional responses to cold stress. Differential gene expression analysis identified four transcriptional clusters. Genes in cluster 1, upregulated in brain but downregulated in gill, were enriched in immune and apoptotic signaling. Cluster 3 genes, conversely upregulated in gill and downregulated in brain, were involved in ion transport and G protein–coupled receptor signaling. Genes in clusters 2 (consistently downregulated) and 4 (consistently upregulated) showed coordinated regulation across both tissues, associated with nucleic acid metabolism, JUN kinase activity, and interferon signaling. These patterns reflect tissue-specific prioritization of physiological functions under cold challenge—immune modulation in brain and ion transport in gill—thereby demonstrating how central hormonal signaling, through mediators such as cortisol, can coordinate peripheral tissue adaptation to maintain systemic homeostasis under stress.

Among the cold-responsive genes, we identified key regulators involved in immune responses, including *pla2g6, cxcl12b, ccl25a*, and *il10*. Genes such as *kcnj11, osbp2*, and *trpc6b* participated in ion transport and membrane excitability. Notably, TRP channel genes (*trpv1, trpa1b, trpc6b*) showed distinct expression dynamics, consistent with their roles as thermosensors and mediators of calcium flux in cold response (Diver et al. 2022). These results suggest that tilapia regulate TRP channel activity to fine-tune calcium signaling and maintain membrane homeostasis under cold conditions.

Chromatin accessibility, a key determinant of transcriptional regulation, was tightly linked to changes in gene expression under cold stress, with approximately two-thirds of differentially expressed genes (DEGs) exhibiting correlated changes in chromatin accessibility. However, the remaining one-third of DEGs showed discordant patterns, indicating that their regulation involves mechanisms beyond proximal promoter accessibility. A plausible explanation involves the role of distal enhancers, which can be located tens to hundreds of kilobases from gene promoters and influence transcription through chromatin looping within topologically associating domains. In such a model, the transcriptional output of a gene could be altered by accessibility changes at these distal regulatory elements, even if the chromatin state of its proximal promoter remains static. Despite this layer of complexity, our analysis successfully identified 96 TFs with significantly enhanced binding activity, including Fra1, Nfil3, Stat1, Mef2d, and Phox2a—many of which are involved in immune regulation and apoptosis (Bell et al. 2024; Jiao et al. 2025; Szeto et al. 2024). This regulatory layer adds important mechanistic insight, demonstrating how environmental cues are transduced through epigenetic modulation of gene expression.

Fra1, a member of the AP-1 transcription factor family, was specifically activated in brain tissue. AP-1 regulates cellular proliferation, apoptosis, and inflammatory signaling and is known to form functional complexes with JunD in fish (Huang et al. 2025). In tilapia, Fra1 targeted genes such as *heatr1, isg15*, and *vcpip1*, which are implicated in ribosomal biogenesis, antiviral responses, and protein homeostasis (Azuma et al. 2006; Huang et al. 2020; Zuo et al. 2024). Enrichment of Fra1 target genes in MAPK, HIF-1, and FoxO signaling pathways further supports its role in orchestrating systemic cold response in the brain.

In contrast, Nrf (Nuclear Respiratory Factor), a CNC-bZIP family member, was preferentially activated in the gill and represents a newly discovered regulator of cold adaptation in teleosts. Nrf targeted genes such as *iscub, hebp2*, and *wdsub1*, which regulate mitochondrial function, oxidative stress responses, and inflammation (Mykkänen et al. 2014; Verleih et al. 2013). Functional enrichment analysis linked Nrf targets to glycine/serine/threonine metabolism, glycerophospholipid biosynthesis, and calcium reabsorption—indicating its central role in maintaining cellular redox balance and membrane integrity in gill tissue under cold stress.

Together, these findings outline a regulatory architecture in which cold stress activates distinct TF modules to mediate adaptive transcriptional programs. Fra1 appears to coordinate stress signaling and neuronal protection in the brain, while Nrf safeguards ion transport and metabolic homeostasis in the gill. This dual-tissue framework reflects the coordinated operation of central neuroendocrine and peripheral physiological systems in coping with environmental perturbations. A substantial subset of the tilapia Fra1- and Nrf-associated target genes possess orthologs across diverse teleost lineages, ranging from polar species such as *Gadus morhua* to tropical species such as *Poecilia reticulata* (Supplementary Table S9). Notably, many of these orthologous loci retain conserved Fra1 or Nrf binding motifs, suggesting that key components of these transcriptional modules are broadly represented across teleosts. At the same time, the relatively modest motif conservation rates and the presence of lineage-specific gains and losses indicate that these networks have undergone considerable evolutionary divergence, likely influenced by motif turnover, gene duplication, and species-specific adaptive pressures. Although our comparative findings support partial conservation of these regulatory architectures, future functional studies in additional fish models will be essential to determine whether Fra1 and Nrf play analogous roles in cold-response pathways across species.

Despite its strengths, this study has several limitations. First, the focus on a single temperature point restricts insights into the temporal dynamics of chromatin remodeling and transcriptional regulation. Future studies employing temperature gradients and time-series sampling could delineate early versus sustained regulatory phases. Second, although our findings highlight Fra1 and Nrf as central regulators in the cold‐response network of Nile tilapia, their applicability as molecular markers for breeding requires further validation. At present, we do not have direct evidence that these genes exhibit consistent expression or sequence differences among tilapia strains with divergent cold-tolerance phenotypes. Our proposal is therefore based on their upstream regulatory positions and broad influence on downstream effectors, which make them biologically plausible candidates for cold-tolerance improvement. To establish their feasibility for molecular breeding, future work should examine natural variation in coding and regulatory regions of Fra1 and Nrf across diverse strains, evaluate genotype–phenotype associations with cold tolerance, and employ targeted functional assays—such as CRISPR-based perturbation or allele-replacement studies—to verify causal effects. These steps will be essential for translating the regulatory insights from this study into practical breeding strategies for developing cold-resilient tilapia lines. Nevertheless, this work provides valuable new perspectives and insights into cold response regulation. Future research should explore the complex interplay between genotype and phenotype more deeply, potentially through multi-omics approaches or refined experimental designs, to yield more comprehensive mechanistic insights.

In summary, this study provides the first multi-omics analysis of brain and gill responses to cold stress in Nile tilapia, revealing tissue-specific transcription factor networks and epigenetic mechanisms of adaptation. By linking chromatin accessibility to gene expression and TF activity, we uncovered a regulatory cascade in which Fra1 and Nrf orchestrate cold-responsive gene expression in distinct tissues. These insights offer a mechanistic foundation for future functional studies and provide molecular targets for improving cold tolerance in aquaculture breeding programs. Our work contributes to the broader understanding of environmental adaptation in ectothermic vertebrates and underscores the importance of integrating chromatin-level data in stress physiology research.

## Conclusion

In summary, this study presents the first integrative epigenomic and transcriptomic analysis of cold stress responses in the brain and gill of Nile tilapia. We identify Fra1 and Nrf as key tissue-specific transcription factors that mediate immune, apoptotic, and metabolic reprogramming under cold exposure. These findings reveal a coordinated regulatory framework linking central and peripheral adaptation through chromatin accessibility and transcriptional control, offering molecular targets for breeding stress-resilient aquaculture strains.

## Materials and methods

### Acute cold stress and sampling

A total of 100 healthy juvenile Nile tilapia (Oreochromis niloticus) with an average weight of 16.67 ± 2.92 g (mean ± SD), sourced from the Center for Aquacultural Breeding Research at Shanghai Ocean University, were used in this study. Prior to the experiment, fish were acclimatized in a recirculating water system maintained at 26–28 °C with dissolved oxygen 6 ± 0.5 mg/L for 7 days, and fed a commercial pellet diet twice daily.

Fish were randomly divided into two groups: control (Ctrl, n = 50) and cold stress (Cold, n = 50). The Cold group was exposed in a 45 L tank containing 25 L of aerated water within an automated cooling chamber (HWS-1000, Jiangnan Instrument Factory, Ningbo, China; temperature range: 0–50 °C, accuracy: ± 0.1 °C). Water temperature was decreased from 28 °C to 10 °C at a rate of 1.5 °C per hour. Control groups were maintained at a constant temperature of 28 °C in the same tank and cooling chamber. Fish were fasted during cooling.

Throughout the experiment, fish behavior was continuously monitored across both groups. No individuals in the Ctrl group exhibited loss of equilibrium (LOE), whereas approximately 60% of individuals in the Cold group showed signs of LOE. Therefore, samples were randomly collected from behaviorally normal individuals in the Ctrl group, and from non-LOE individuals in the Cold group to ensure consistency in physiological status for downstream analyses.

After 12 h of cooling, surviving fish were randomly selected for euthanasia with isoflurane MS-222 (100 mg·L⁻^1^; West Gene, #886–86-2). Brain and gill tissues were snap-frozen for integrated RNA-seq and ATAC-seq analyses.

#### Cortisol extraction and quantification

Cortisol levels in plasma and brain tissue were quantified using a commercial cortisol ELISA kit (mlbio). Blood samples were collected and allowed to clot at room temperature, followed by centrifugation at 4000 rpm for 20 min to obtain plasma. Brain tissues were homogenized in phosphate-buffered saline (BBI #E607008-0500) and the supernatant was collected after centrifugation. The assay was performed strictly according to the manufacturer's protocol. The optical density was measured at 450 nm using a microplate reader, and cortisol concentrations were determined by interpolation from the standard curve included in each assay run.

#### ATAC-seq

For ATAC-seq, nuclei from brain and gill were isolated following Grandi et al. (Grandi et al. 2022). Nuclei were centrifuged at 500 × g for 10 min at 4 °C, after which the supernatant was discarded and the pellet resuspended in 50 µL tagmentation mix (Illumina #20,034,210) containing 25 µL 2 × TD buffer, 16.5 µL 1 × PBS, 0.5 µL 10% Tween-20, 0.5 µL 1% Digitonin, 2.5 µL Tn5 transposase, and 5 µL nuclease-free H₂O. Tagmentation proceeded at 37 °C for 30 min in a thermomixer (Eppendorf Thermomixer Comfort) at 1000 rpm. Tagmented DNA was purified using the MinElute Reaction Cleanup Kit (QIAGEN #28204, China), with fragment quality assessed by agarose gel electrophoresis. PCR amplification and purification preceded library quantification via Qubit (Invitrogen) and size distribution analysis on an Agilent 2100 Bioanalyzer. Final libraries were sequenced on Illumina NovaSeq 6000. Eight ATAC-seq libraries were generated with two biological replicates per tissue-temperature combination: brain and gill tissues under both 28 °C (control) and 10 °C (cold stress) conditions.

#### RNA-seq

For RNA-seq, total RNA was extracted with Trizol reagent (Invitrogen #10296010CN) per manufacturer's protocol. RNA integrity was verified by 1.5% agarose gel electrophoresis, while purity and concentration were determined by NanoDrop. From 1 µg total RNA per sample, libraries were prepared using the Hieff NGS® Ultima Dual-mode mRNA Library Prep Kit (YEASEN #12,309), executing mRNA isolation, fragmentation, double-stranded cDNA synthesis, end repair, A-tailing, adapter ligation, and library amplification to ensure compatibility with Illumina platforms. The libraries were quantified with a Qubit 4 fluorometer (Invitrogen) and assessed using the Agilent Bioanalyzer 2100, followed by sequencing on the Illumina NovaSeq 6000 platform with three biological replicates for RNA-seq.

#### Integration analysis of RNA-seq and ATAC-seq

We generated 20 new datasets (12 RNA-seq, 8 ATAC-seq) from tilapia brain and gill tissues. For RNA-seq analysis, raw reads were adapter-trimmed and quality-filtered using fastp (v0.23.4) (Chen et al. 2018) before alignment to the *Oreochromis niloticus* reference genome (O_niloticus_UMD_NMBU, GCA_001858045.3; downloaded from https://ftp.ensembl.org/pub/release-109/fasta/oreochromis_niloticus/dna/Oreochromis_niloticus.O_niloticus_UMD_NMBU.dna.toplevel.fa.gz) via HISAT2 (v2.2.1) (Kim et al. 2019). Low-quality bases (Q < 15) were trimmed and adapter sequences were removed to ensure high-quality reads for downstream analysis. The reference genome index used for RNA-seq data alignment was constructed using the hisat2-build function from the HISAT2 software. Aligned reads were sorted/converted to BAM files using samtools (v1.18) (Danecek et al. 2021), with gene-level quantification performed by featureCounts (v2.0.6) (Liao et al. 2014). Transcript-per-million (TPM) values were calculated from counts using custom R scripts (v4.4.1). Principal component analysis utilized variance-stabilized transformed counts in DESeq2 (v1.44.0) (Fig. 1C) (Love et al. 2014). Differentially expressed genes (DEGs) were identified with DESeq2 (|log₂FC|> 1, p < 0.05), followed by k-means clustering of Z-scaled TPM values and visualization via ComplexHeatmap (v2.20.0) (Fig. 1D) (Gu et al. 2016).

ATAC-seq reads were processed with fastp (v0.23.4; –detect_adapter_for_pe), aligned using BWA-MEM (v0.7.18) to the same reference genome, and filtered (MAPQ ≥ 30, samtools -F 1804 -f 2). The reference genome index used for ATAC-seq data alignment was constructed using the bwa index function from the BWA-MEM soft. PCR duplicates were removed via Picard (v3.1.1; REMOVE_DUPLICATES = true). Replicate BAM files were merged (samtools merge), converted to bigWig with deepTools (v3.5.4; bamCoverage –normalizeUsing RPKM –binSize 10 –effectiveGenomeSize 1,005,681,550) (Ramírez et al. 2014), and visualized in IGV (v2.18.0) (Fig. 1H). MACS2 (v2.2.7.1; -q 0.05 -f BAMPE –nomodel –extsize 200 –shift −100) called peaks (Zhang et al. 2008), which were merged/quantified by DiffBind (v3.14.0; bUseSummarizeOverlaps = TRUE) (Stark and Brown, 2012). Peaks were annotated with ChIPseeker (v1.40.0) as promoter (−3 kb to + 500 bp from TSS) (Yu et al. 2015), genic (+ 500 bp to TES), or distal regions (Fig. S4B). Differentially accessible peaks (DAPs) were identified from count-per-million (CPM) values using DESeq2. Peaks located in the genomic regions of DEGs and exhibiting accessibility trends consistent with the temperature-dependent expression patterns of the corresponding DEGs were defined as DAPs (Differentially Accessible Peaks). Peak-gene associations required matched expression/accessibility patterns (Z-scaled TPM vs. TFBS) across replicates (Fig. 3C). TSS signal profiles were computed with deepTools computeMatrix/plotProfile (Fig. 2B, S5).

#### TF annotation, motif calling and enrichment

Tilapia transcription factors (TFs) were classified into families using AnimalTFDB4 (https://guolab.wchscu.cn/AnimalTFDB4/#/). TF family enrichment analysis for DEGs was conducted via the enricher function in clusterProfiler (v4.12.5) (Fig. 1F) (Wu et al. 2021). Footprint analysis was performed with TOBIAS (v0.14.0) (Beisaw et al. 2020) using HOMER motif databases (Fig. 3D) (Heinz et al. 2010). De novo motif discovery executed HOMER's findMotifsGenome.pl program, with known motif matches identified via annotatePeaks.pl.

Protein sequences were annotated via eggNOG (v4.5) (Powell et al. 2014) for GO/KEGG terms, with enrichment tested by clusterProfiler (v4.12.5) (Figs. 1G, 2C).

#### Transcriptional regulatory network construction

Following Liu et al. (Liu et al. 2023), we integrated DEGs (p < 0.05, |log₂FC|> 1) with promoter/genic ATAC-seq peaks. TOBIAS identified footprint-motif pairs to establish TF-target relationships. Resulting networks (TF-TF and TF-target edges) were built with TOBIAS CreateNetwork and visualized in Cytoscape (v3.10.3) (https://cytoscape.org/) (Figs. 4E, 5E, S6) (Cline et al. 2007).

#### HEK293T cell-based calcium imaging

The open reading frames (ORFs) of tilapia *trpv1* were synthesized by GENEWIZ and subsequently amplified. The fragments were sequenced and ligated into the pcDNA3.1 vector using Gibson assembly (NEB #E2621). Calcium imaging in HEK293T cells was performed using genetically encoded calcium indicators (GCaMPs) to monitor intracellular Ca^2+^ dynamics, as previously described (Su et al. 2024). Briefly, HEK293T cells were transfected with pLVX plasmids carrying the GCaMP6s gene, along with lentiviral packaging elements, using Lipofectamine 2000 (Invitrogen). Lentiviral particles were harvested 48–72 h after transfection, followed by purification and concentration. To establish a stable GCaMP6s-expressing cell line, HEK293T cells were infected with 2 μL of concentrated lentivirus and subjected to puromycin selection. The culture medium was replaced every two days over a period of two weeks, until resistant cell clones emerged. One day prior to imaging, the GCaMP6s-expressing cells were seeded onto 35-mm Mattek dishes. These cells were then transfected with either a negative control (NC) or wild-type *trpv1* constructs using Lipofectamine 2000. Calcium signals were recorded two days post-transfection at 10 °C using an Axio Observer Z1 microscope (Zeiss), capturing fluorescence images at 5-s intervals to assess baseline calcium activity (see Supplemental Videos 1–2). To evoke Ca^2+^ influx, 0.5 μM capsaicin (Cap) was applied, and changes in intracellular calcium levels were detected via the GCaMP6s sensor expressed in HEK293T cells.

#### Experimental validation of transcription factor function to cold response

To validate the roles of the identified motifs, upstream promoter sequences containing the selected motifs with the proper lengths from the target genes were synthesized de novo by GENEWIZ. For cell transfection, the tested wild-type promoter sequences were cloned into the pGL4.23 vector. The corresponding motif mutants were created by directly synthesizing the promoter sequences in which the nucleotides of the wild-type motif were replaced with an equal number of thymidines. All constructed plasmids were verified by DNA sequencing.

EPC cells were maintained in DMEM medium supplemented with 10% FBS at 28 °C under 5% CO_2_. Plasmid DNAs were transiently transfected into EPC cells using the jetPRIME transfection reagent (Polyplus) following the manufacturer's protocol. Briefly, a mixture of 0.8 μg of the pGL4.23 plasmid carrying the wild-type or mutated promoter sequence and 0.2 μg of the pRL-TK control plasmid was transfected into cells seeded in a 12-well plate. Eight hours after transfection, the cells were transferred to a low-temperature incubator (ESCO) and exposed to a temperature of 23 °C for 24 h. After the cold treatment, cells maintained at 28 °C and 23 °C were harvested for total RNA isolation. Total RNA (1 μg) was reverse-transcribed into cDNA using ABScript Neo RT Master Mix for qPCR (ABclonal). Quantitative PCR (qPCR) was then performed using the SsoFast™ EvaGreen® Supermix (Bio-Rad) according to the manufacturer's instructions. The expression levels of firefly luciferase were normalized to those of Renilla luciferase. All assays were conducted with three technical replicates and independently repeated three times. The primers used for qPCR are listed in Supplementary Table S8.

## Supplementary Information

Supplementary Material 1: Fig. S1. Shared and tissue-specific transcriptional responses to cold stress in tilapia brain and gill tissues. (A) Pairwise correlation analysis of RNA-seq replicates across experimental conditions (Brain/Gill, Cold/Ctrl), presented as a hierarchical clustering heatmap. (B) Venn diagram illustrating the overlap of DEGs(left) and DAPs (right) between Brain and Gill tissues under cold stress. (C) Comparative analysis of differentially expressed genes (DEGs) between Brain and Gill tissues under Cold and Ctrl groups, visualized through Venn diagrams. (D) Volcano plots identifying significant transcriptional changes in Brain (left) and Gill (right) tissues during cold exposure, plotted by fold-change versus statistical significance. (E) Pathway enrichment analysis (KEGG) of common and tissue-specific DEGs in response to cold stress. KEGG, kyoto encyclopedia of genes and genomes; Cold, cold; Ctrl, control; DEGs, differentially expressed genes; DAPs, differentially accessible peaks. Fig. S2. Functional validation of tilapia TRPV1 channel using real-time Ca²⁺ imaging. (A) Real-time intracellular Ca²⁺ dynamics in GCaMP6-stable HEK293T cells transfected with a negative control (NC) plasmid or a plasmid encoding tilapia trpv1. Cells were treated with 0.5 μM capsaicin (Cap), a specific TRPV1 agonist, and Ca²⁺ signals were recorded at low temperature. The y-axis represents the normalized fluorescence intensity (F/F₀) of GCaMP6, reflecting intracellular Ca²⁺ concentration. (B) Quantitative analysis of the frequency of Ca²⁺ transients in NC and TRPV1-overexpressing cells. Each point represents one field of view from an independent dish. Statistical significance was assessed using an unpaired two-tailed *t*-test. Fig. S3. Library quality assessment for chromatin accessibility profiling. Distribution of ATAC-seq fragment sizes across experimental conditions, comparing Brain and Gill tissues under both Ctrl and Cold treatments. Data represent two independent biological replicates per condition. Cold, cold; Ctrl, control. Fig. S4. Inter-sample relationships and chromatin landscape characterization in tilapia. (A) Pairwise correlation analysis of ATAC-seq replicates across experimental groups (Brain/Gill, Cold/Ctrl), presented as a clustered heatmap. (B) Genomic annotation of chromatin accessibility peaks, showing their distribution across functional regions in Brain and Gill tissues. Cold, cold; Ctrl, control. Fig. S5. Cold stress-induced chromatin accessibility changes in Brain and Gill tissues of tilapia. Chromatin accessibility profiles of 5,614 differentially expressed genes (DEGs) near transcription start sites (TSS) in brain tissue under cold exposure. Corresponding chromatin dynamics patterns for DEG-associated TSS regions in gill tissue during cold stress. DEGs: differentially expressed genes; TSS: transcription start site. Fig. S6. Transcriptional regulatory networks modulated by cold stress. (A) Brain tissue regulatory network integrating gene expression profiles, chromatin accessibility data, and transcription factor (TF) binding information during cold exposure. (B) Corresponding regulatory network in Gill tissue under cold stress, showing only TFs with functional interactions. Node coloration reflects expression changes relative to control conditions.

Supplementary Material 2.

Supplementary Material 3.

Supplementary Material 4.

Supplementary Material 5.

## Data Availability

All data are presented in the figures and tables or in the electronic supplementary material, figures and tables. The sequencing data for this study are available in the BIG Sub Genome Sequence Archive at the China National Genomics Data Center database under accession no. CRA027381.

## Abbreviations

ATAC-seq: Assay for Transposase-Accessible Chromatin using sequencing
RNA-seq: RNA sequencing
TF: Transcription Factor
DEG: Differentially Expressed Gene
DAP: Differentially Accessible Peak
TRN: Transcriptional Regulatory Network
PCA: Principal Component Analysis
TPM: Transcripts Per Million
GO: Gene Ontology
KEGG: Kyoto Encyclopedia of Genes and Genomes
AP-1: Activator Protein-1
ARE: Antioxidant Response Element
IGV: Integrative Genomics Viewer
Hi-C: High-throughput Chromosome Conformation Capture
LOE: Loss of Equilibrium
TSS: Transcription Start Site
TES: Transcription End Site
bp: Base pair
SD: Standard Deviation
