# Supplementary figures and images for: Integrative epigenomic and transcriptomic profiling reveals organ-specific and coordinated cold stress responses in the brain and gill of Nile tilapia
Source: Stress Biol. 2026 Jan 11;6(1):4. doi: 10.1007/s44154-025-00277-y (PMC12790559; doi:10.1007/s44154-025-00277-y)

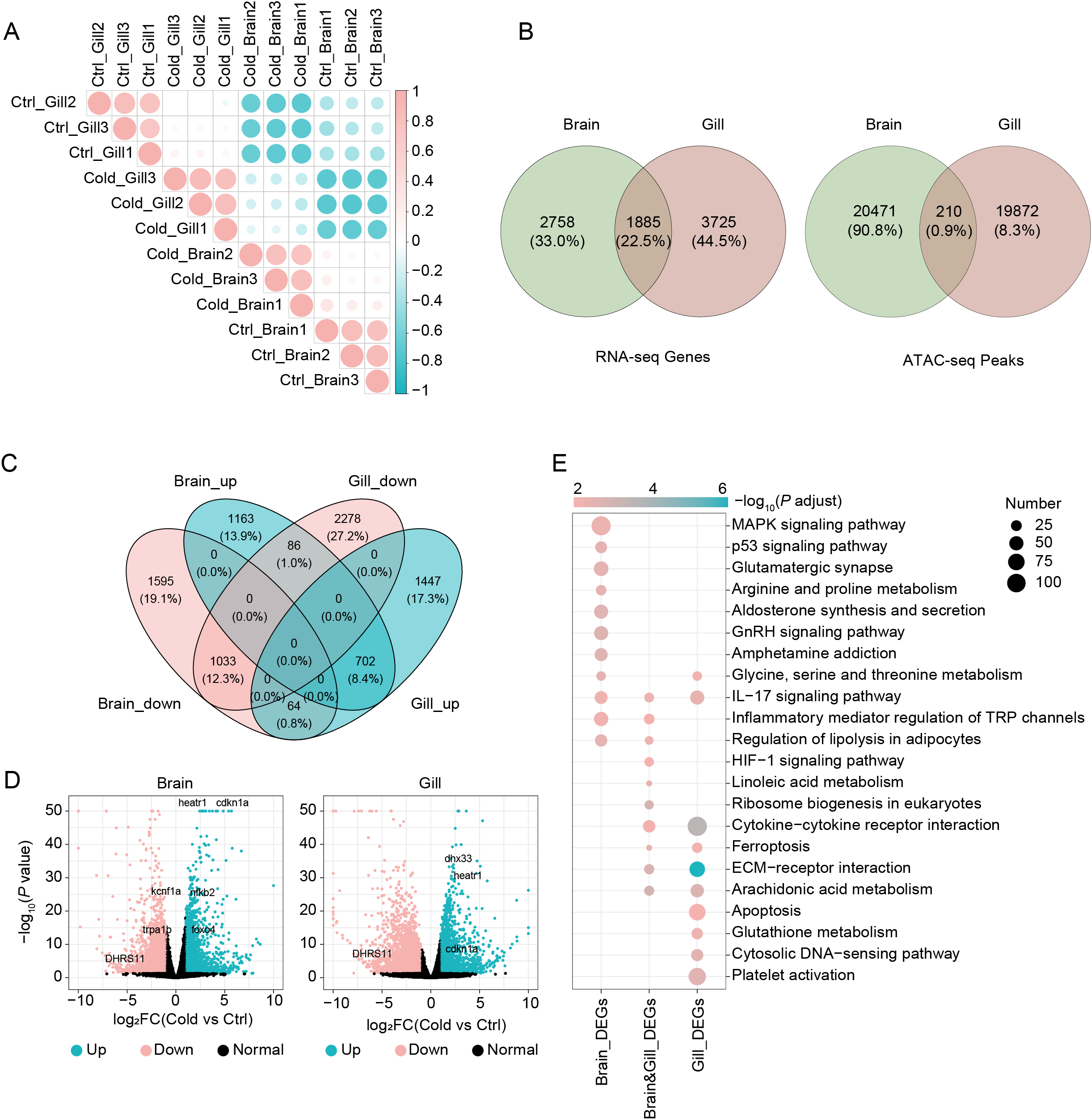

Supplement: Supplementary file 1 — Supplementary Material 1: Fig. S1. Shared and tissue-specific transcriptional responses to cold stress in tilapia brain and gill tissues. (A) Pairwise correlation analysis of RNA-seq replicates across experimental conditions (Brain/Gill, Cold/Ctrl), presented as a hierarchical clustering heatmap. (B) Venn diagram illustrating the overlap of DEGs(left) and DAPs (right) between Brain and Gill tissues under cold stress. (C) Comparative analysis of differentially expressed genes (DEGs) between Brain and Gill tissues under Cold and Ctrl groups, visualized through Venn diagrams. (D) Volcano plots identifying significant transcriptional changes in Brain (left) and Gill (right) tissues during cold exposure, plotted by fold-change versus statistical significance. (E) Pathway enrichment analysis (KEGG) of common and tissue-specific DEGs in response to cold stress. KEGG, kyoto encyclopedia of genes and genomes; Cold, cold; Ctrl, control; DEGs, differentially expressed genes; DAPs, differentially accessible peaks. Fig. S2. Functional validation of tilapia TRPV1 channel using real-time Ca²⁺ imaging. (A) Real-time intracellular Ca²⁺ dynamics in GCaMP6-stable HEK293T cells transfected with a negative control (NC) plasmid or a plasmid encoding tilapia trpv1. Cells were treated with 0.5 μM capsaicin (Cap), a specific TRPV1 agonist, and Ca²⁺ signals were recorded at low temperature. The y-axis represents the normalized fluorescence intensity (F/F₀) of GCaMP6, reflecting intracellular Ca²⁺ concentration. (B) Quantitative analysis of the frequency of Ca²⁺ transients in NC and TRPV1-overexpressing cells. Each point represents one field of view from an independent dish. Statistical significance was assessed using an unpaired two-tailed t-test. Fig. S3. Library quality assessment for chromatin accessibility profiling. Distribution of ATAC-seq fragment sizes across experimental conditions, comparing Brain and Gill tissues under both Ctrl and Cold treatments. Data represent two independent [file 44154_2025_277_MOESM1_ESM.zip › Supplementary Figures/figureS1R1.png]

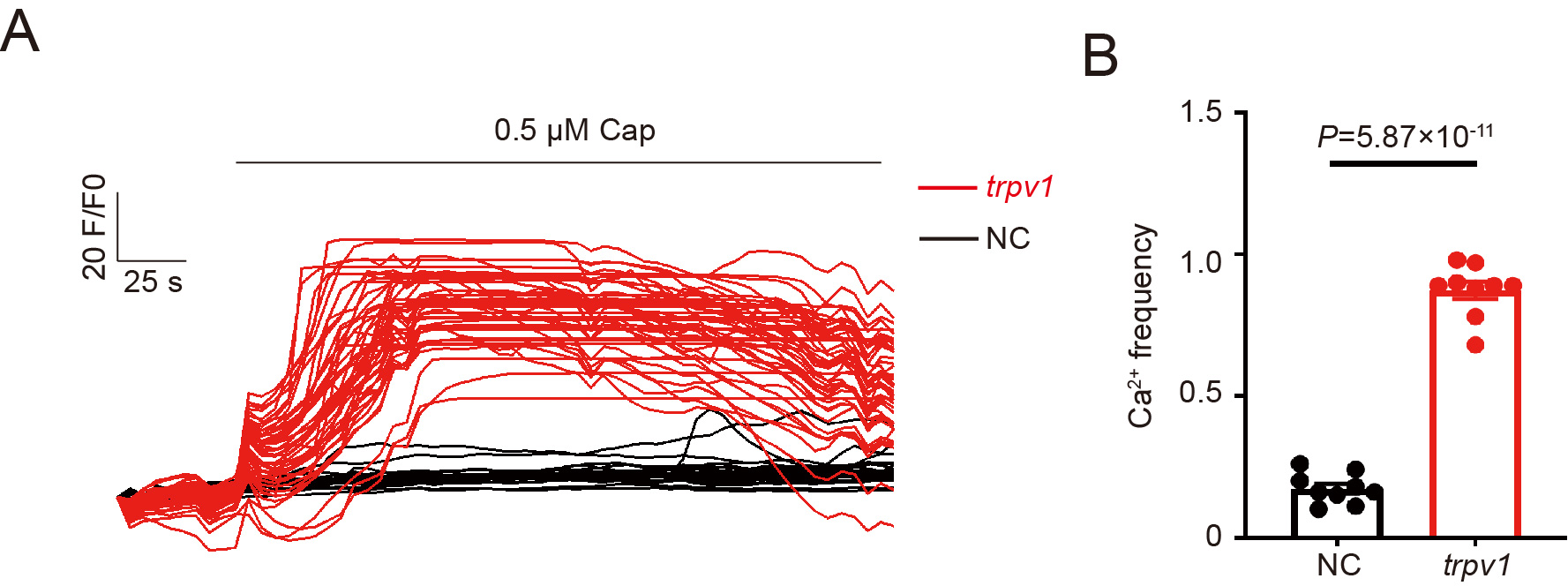

Supplement: Supplementary file 1 — Supplementary Material 1: Fig. S1. Shared and tissue-specific transcriptional responses to cold stress in tilapia brain and gill tissues. (A) Pairwise correlation analysis of RNA-seq replicates across experimental conditions (Brain/Gill, Cold/Ctrl), presented as a hierarchical clustering heatmap. (B) Venn diagram illustrating the overlap of DEGs(left) and DAPs (right) between Brain and Gill tissues under cold stress. (C) Comparative analysis of differentially expressed genes (DEGs) between Brain and Gill tissues under Cold and Ctrl groups, visualized through Venn diagrams. (D) Volcano plots identifying significant transcriptional changes in Brain (left) and Gill (right) tissues during cold exposure, plotted by fold-change versus statistical significance. (E) Pathway enrichment analysis (KEGG) of common and tissue-specific DEGs in response to cold stress. KEGG, kyoto encyclopedia of genes and genomes; Cold, cold; Ctrl, control; DEGs, differentially expressed genes; DAPs, differentially accessible peaks. Fig. S2. Functional validation of tilapia TRPV1 channel using real-time Ca²⁺ imaging. (A) Real-time intracellular Ca²⁺ dynamics in GCaMP6-stable HEK293T cells transfected with a negative control (NC) plasmid or a plasmid encoding tilapia trpv1. Cells were treated with 0.5 μM capsaicin (Cap), a specific TRPV1 agonist, and Ca²⁺ signals were recorded at low temperature. The y-axis represents the normalized fluorescence intensity (F/F₀) of GCaMP6, reflecting intracellular Ca²⁺ concentration. (B) Quantitative analysis of the frequency of Ca²⁺ transients in NC and TRPV1-overexpressing cells. Each point represents one field of view from an independent dish. Statistical significance was assessed using an unpaired two-tailed t-test. Fig. S3. Library quality assessment for chromatin accessibility profiling. Distribution of ATAC-seq fragment sizes across experimental conditions, comparing Brain and Gill tissues under both Ctrl and Cold treatments. Data represent two independent [file 44154_2025_277_MOESM1_ESM.zip › Supplementary Figures/figureS2R1.jpg]

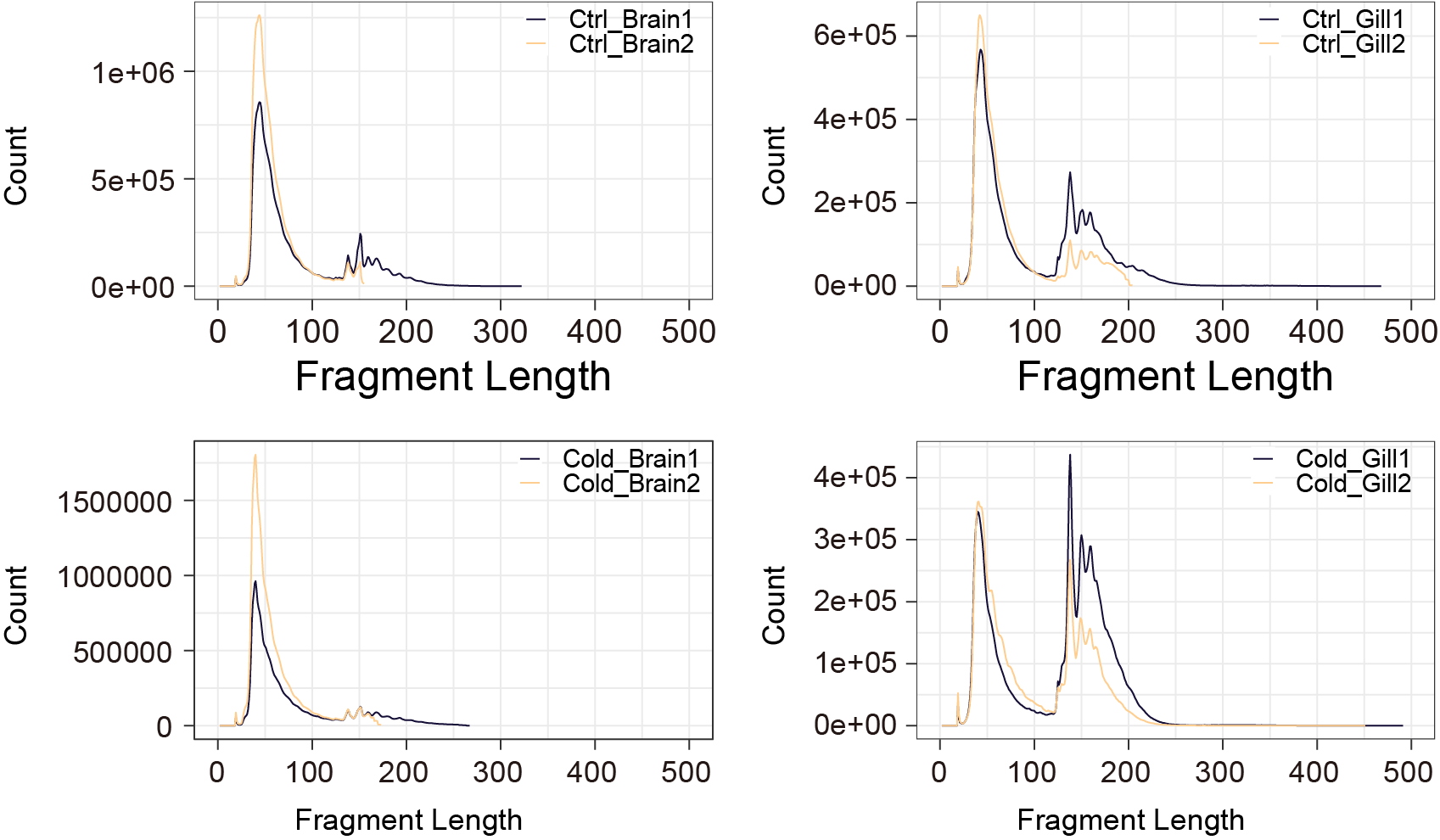

Supplement: Supplementary file 1 — Supplementary Material 1: Fig. S1. Shared and tissue-specific transcriptional responses to cold stress in tilapia brain and gill tissues. (A) Pairwise correlation analysis of RNA-seq replicates across experimental conditions (Brain/Gill, Cold/Ctrl), presented as a hierarchical clustering heatmap. (B) Venn diagram illustrating the overlap of DEGs(left) and DAPs (right) between Brain and Gill tissues under cold stress. (C) Comparative analysis of differentially expressed genes (DEGs) between Brain and Gill tissues under Cold and Ctrl groups, visualized through Venn diagrams. (D) Volcano plots identifying significant transcriptional changes in Brain (left) and Gill (right) tissues during cold exposure, plotted by fold-change versus statistical significance. (E) Pathway enrichment analysis (KEGG) of common and tissue-specific DEGs in response to cold stress. KEGG, kyoto encyclopedia of genes and genomes; Cold, cold; Ctrl, control; DEGs, differentially expressed genes; DAPs, differentially accessible peaks. Fig. S2. Functional validation of tilapia TRPV1 channel using real-time Ca²⁺ imaging. (A) Real-time intracellular Ca²⁺ dynamics in GCaMP6-stable HEK293T cells transfected with a negative control (NC) plasmid or a plasmid encoding tilapia trpv1. Cells were treated with 0.5 μM capsaicin (Cap), a specific TRPV1 agonist, and Ca²⁺ signals were recorded at low temperature. The y-axis represents the normalized fluorescence intensity (F/F₀) of GCaMP6, reflecting intracellular Ca²⁺ concentration. (B) Quantitative analysis of the frequency of Ca²⁺ transients in NC and TRPV1-overexpressing cells. Each point represents one field of view from an independent dish. Statistical significance was assessed using an unpaired two-tailed t-test. Fig. S3. Library quality assessment for chromatin accessibility profiling. Distribution of ATAC-seq fragment sizes across experimental conditions, comparing Brain and Gill tissues under both Ctrl and Cold treatments. Data represent two independent [file 44154_2025_277_MOESM1_ESM.zip › Supplementary Figures/figureS3R1.png]

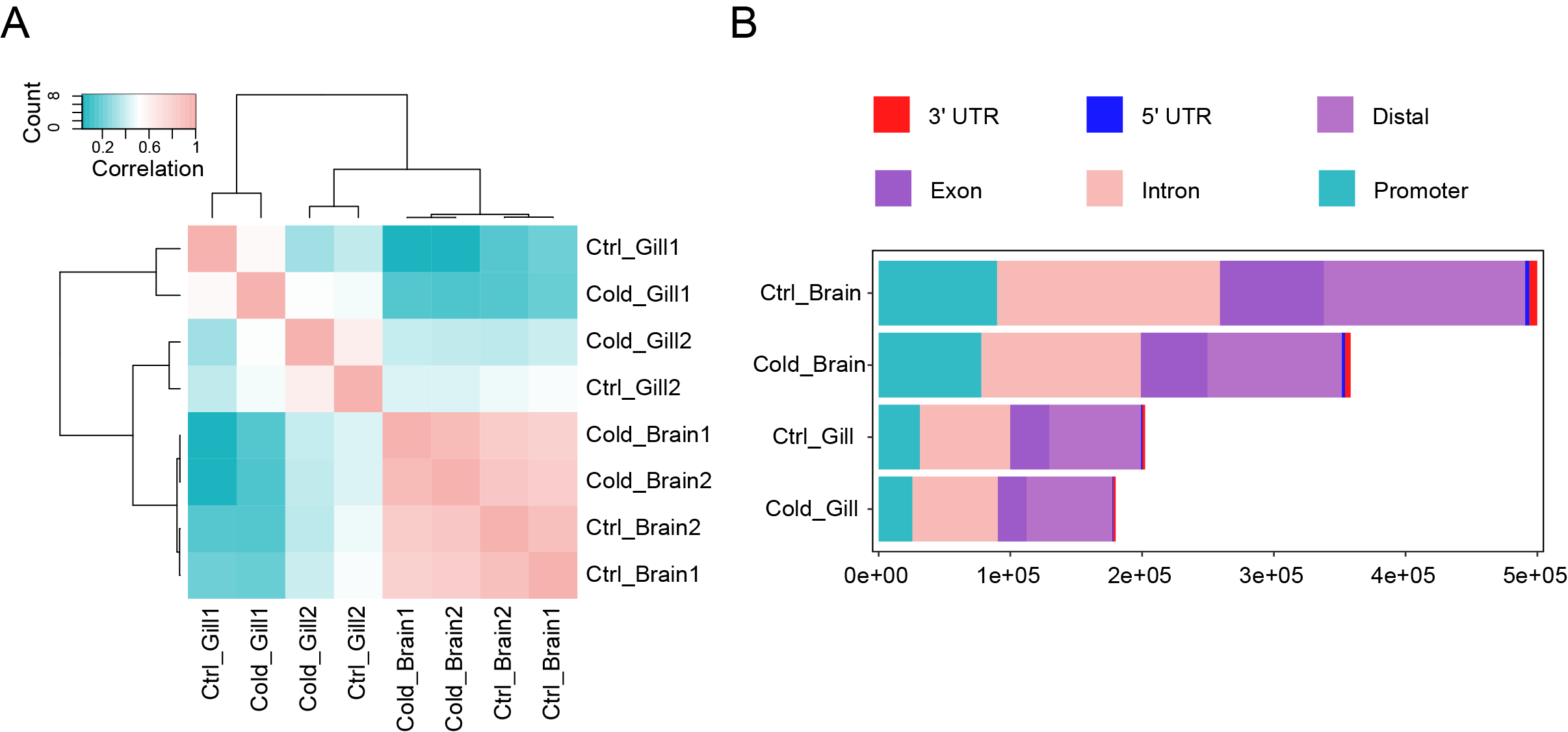

Supplement: Supplementary file 1 — Supplementary Material 1: Fig. S1. Shared and tissue-specific transcriptional responses to cold stress in tilapia brain and gill tissues. (A) Pairwise correlation analysis of RNA-seq replicates across experimental conditions (Brain/Gill, Cold/Ctrl), presented as a hierarchical clustering heatmap. (B) Venn diagram illustrating the overlap of DEGs(left) and DAPs (right) between Brain and Gill tissues under cold stress. (C) Comparative analysis of differentially expressed genes (DEGs) between Brain and Gill tissues under Cold and Ctrl groups, visualized through Venn diagrams. (D) Volcano plots identifying significant transcriptional changes in Brain (left) and Gill (right) tissues during cold exposure, plotted by fold-change versus statistical significance. (E) Pathway enrichment analysis (KEGG) of common and tissue-specific DEGs in response to cold stress. KEGG, kyoto encyclopedia of genes and genomes; Cold, cold; Ctrl, control; DEGs, differentially expressed genes; DAPs, differentially accessible peaks. Fig. S2. Functional validation of tilapia TRPV1 channel using real-time Ca²⁺ imaging. (A) Real-time intracellular Ca²⁺ dynamics in GCaMP6-stable HEK293T cells transfected with a negative control (NC) plasmid or a plasmid encoding tilapia trpv1. Cells were treated with 0.5 μM capsaicin (Cap), a specific TRPV1 agonist, and Ca²⁺ signals were recorded at low temperature. The y-axis represents the normalized fluorescence intensity (F/F₀) of GCaMP6, reflecting intracellular Ca²⁺ concentration. (B) Quantitative analysis of the frequency of Ca²⁺ transients in NC and TRPV1-overexpressing cells. Each point represents one field of view from an independent dish. Statistical significance was assessed using an unpaired two-tailed t-test. Fig. S3. Library quality assessment for chromatin accessibility profiling. Distribution of ATAC-seq fragment sizes across experimental conditions, comparing Brain and Gill tissues under both Ctrl and Cold treatments. Data represent two independent [file 44154_2025_277_MOESM1_ESM.zip › Supplementary Figures/figureS4R1.png]

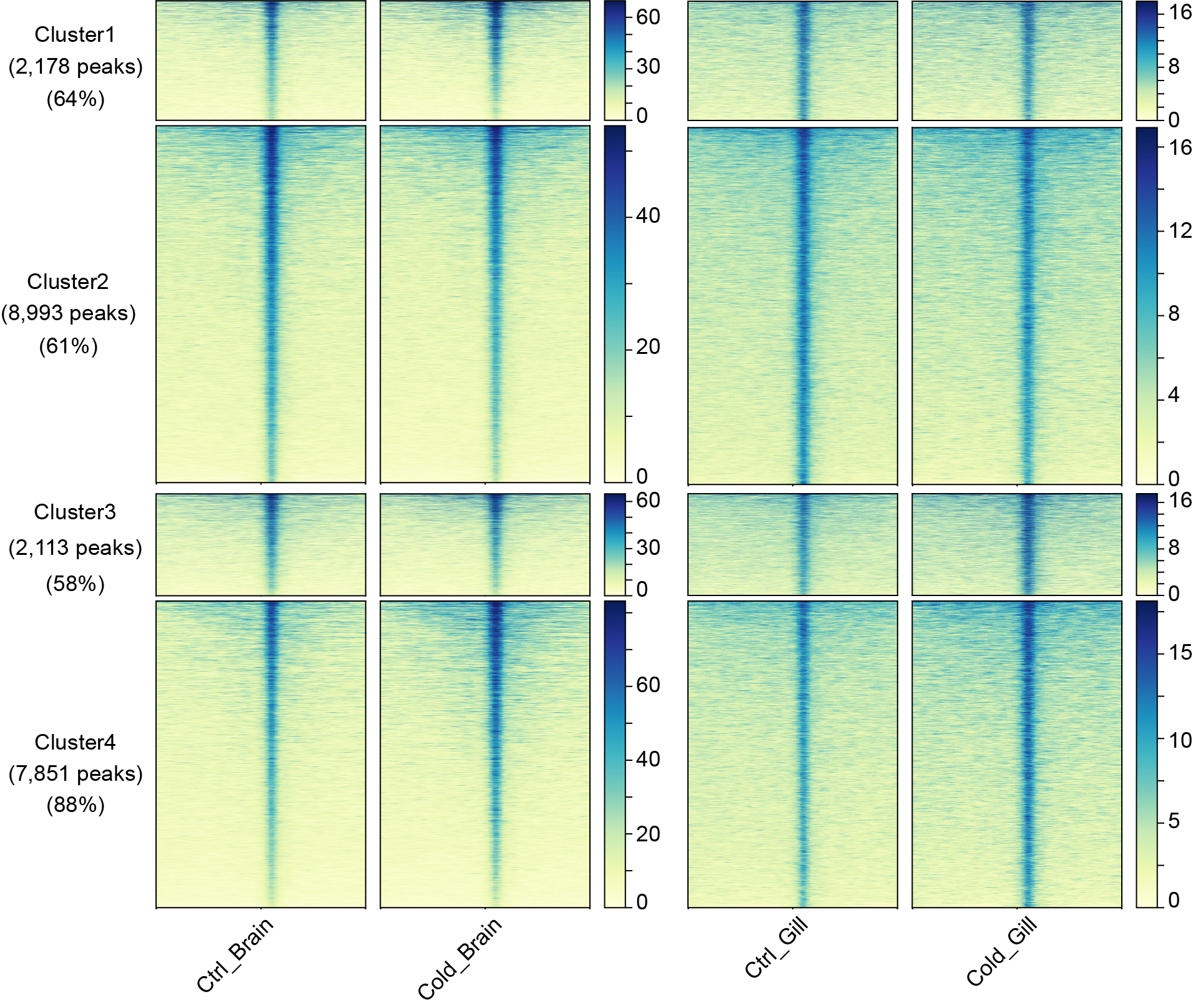

Supplement: Supplementary file 1 — Supplementary Material 1: Fig. S1. Shared and tissue-specific transcriptional responses to cold stress in tilapia brain and gill tissues. (A) Pairwise correlation analysis of RNA-seq replicates across experimental conditions (Brain/Gill, Cold/Ctrl), presented as a hierarchical clustering heatmap. (B) Venn diagram illustrating the overlap of DEGs(left) and DAPs (right) between Brain and Gill tissues under cold stress. (C) Comparative analysis of differentially expressed genes (DEGs) between Brain and Gill tissues under Cold and Ctrl groups, visualized through Venn diagrams. (D) Volcano plots identifying significant transcriptional changes in Brain (left) and Gill (right) tissues during cold exposure, plotted by fold-change versus statistical significance. (E) Pathway enrichment analysis (KEGG) of common and tissue-specific DEGs in response to cold stress. KEGG, kyoto encyclopedia of genes and genomes; Cold, cold; Ctrl, control; DEGs, differentially expressed genes; DAPs, differentially accessible peaks. Fig. S2. Functional validation of tilapia TRPV1 channel using real-time Ca²⁺ imaging. (A) Real-time intracellular Ca²⁺ dynamics in GCaMP6-stable HEK293T cells transfected with a negative control (NC) plasmid or a plasmid encoding tilapia trpv1. Cells were treated with 0.5 μM capsaicin (Cap), a specific TRPV1 agonist, and Ca²⁺ signals were recorded at low temperature. The y-axis represents the normalized fluorescence intensity (F/F₀) of GCaMP6, reflecting intracellular Ca²⁺ concentration. (B) Quantitative analysis of the frequency of Ca²⁺ transients in NC and TRPV1-overexpressing cells. Each point represents one field of view from an independent dish. Statistical significance was assessed using an unpaired two-tailed t-test. Fig. S3. Library quality assessment for chromatin accessibility profiling. Distribution of ATAC-seq fragment sizes across experimental conditions, comparing Brain and Gill tissues under both Ctrl and Cold treatments. Data represent two independent [file 44154_2025_277_MOESM1_ESM.zip › Supplementary Figures/figureS5R1.png]

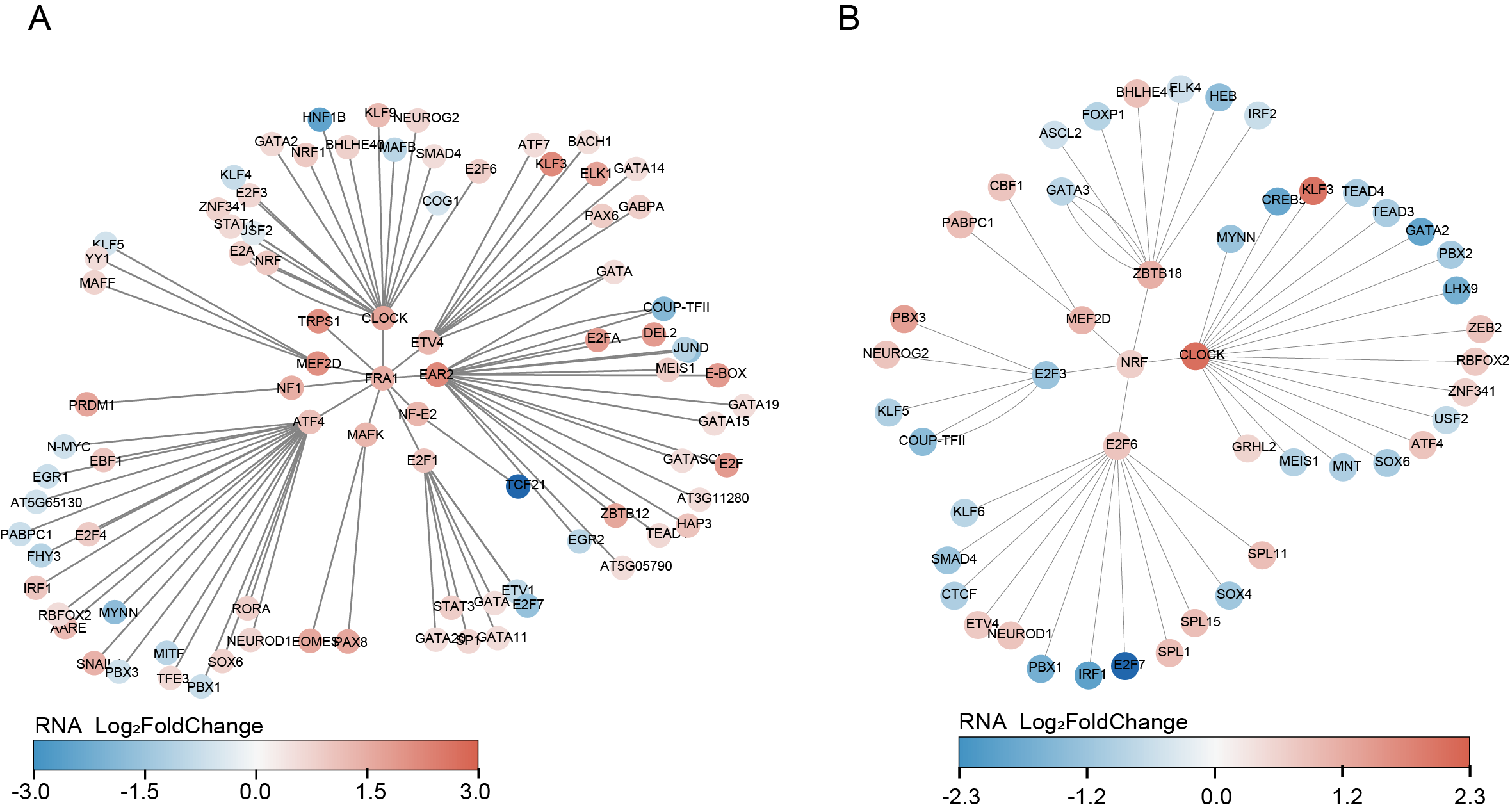

Supplement: Supplementary file 1 — Supplementary Material 1: Fig. S1. Shared and tissue-specific transcriptional responses to cold stress in tilapia brain and gill tissues. (A) Pairwise correlation analysis of RNA-seq replicates across experimental conditions (Brain/Gill, Cold/Ctrl), presented as a hierarchical clustering heatmap. (B) Venn diagram illustrating the overlap of DEGs(left) and DAPs (right) between Brain and Gill tissues under cold stress. (C) Comparative analysis of differentially expressed genes (DEGs) between Brain and Gill tissues under Cold and Ctrl groups, visualized through Venn diagrams. (D) Volcano plots identifying significant transcriptional changes in Brain (left) and Gill (right) tissues during cold exposure, plotted by fold-change versus statistical significance. (E) Pathway enrichment analysis (KEGG) of common and tissue-specific DEGs in response to cold stress. KEGG, kyoto encyclopedia of genes and genomes; Cold, cold; Ctrl, control; DEGs, differentially expressed genes; DAPs, differentially accessible peaks. Fig. S2. Functional validation of tilapia TRPV1 channel using real-time Ca²⁺ imaging. (A) Real-time intracellular Ca²⁺ dynamics in GCaMP6-stable HEK293T cells transfected with a negative control (NC) plasmid or a plasmid encoding tilapia trpv1. Cells were treated with 0.5 μM capsaicin (Cap), a specific TRPV1 agonist, and Ca²⁺ signals were recorded at low temperature. The y-axis represents the normalized fluorescence intensity (F/F₀) of GCaMP6, reflecting intracellular Ca²⁺ concentration. (B) Quantitative analysis of the frequency of Ca²⁺ transients in NC and TRPV1-overexpressing cells. Each point represents one field of view from an independent dish. Statistical significance was assessed using an unpaired two-tailed t-test. Fig. S3. Library quality assessment for chromatin accessibility profiling. Distribution of ATAC-seq fragment sizes across experimental conditions, comparing Brain and Gill tissues under both Ctrl and Cold treatments. Data represent two independent [file 44154_2025_277_MOESM1_ESM.zip › Supplementary Figures/figureS6R1.png]
